# Effect of sodium triphosphate on particle size of heat‐induced whey protein concentrate aggregates

**DOI:** 10.1002/fsn3.665

**Published:** 2018-09-07

**Authors:** Diru Liu, Jianjun Cheng, Changhui Zhao, Mingruo Guo

**Affiliations:** ^1^ School of Public Health Lanzhou University Lanzhou China; ^2^ College of Food Science Northeast Agricultural University Harbin China; ^3^ College of Food Science and Engineering Jilin University Changchun China; ^4^ Department of Nutrition and Food Sciences College of Agriculture and Life Sciences University of Vermont Burlington Vermont

**Keywords:** fat replacer, particle size distribution, sodium triphosphate, thermal treatment, whey protein concentrate

## Abstract

Thermal treatment has been utilized to improve the functional properties of proteins for many years. In this study, we aimed to investigate the effect of sodium triphosphate (Na_5_P_3_O_10_) on particle size and size distribution of heat‐induced whey protein concentrate (WPC) aggregates under different processing conditions. The results showed that high Na_5_P_3_O_10_ level (>0.5%, w/w), long heating time (>15 min), and alkaline condition (pH 8–8.5) facilitated formation of large particles (>10 μm). The WPC aggregates with small‐to‐medium particle size (1–3 μm) that are suitable to be applied as a fat replacer were obtained by heating the WPC solution (8%, w/v) containing 0.4% (w/w) Na_5_P_3_O_10_ at 85°C for 5 min. We conclude that thermal treatment of whey protein concentrate added with Na_5_P_3_O_10_ can obtain whey protein products with different particle sizes for certain applications.

## 1 INTRODUCTION

Whey proteins (WP) have been widely used in various food products, including meat, snacks, drinks, and dairy products, because whey proteins have diverse functional properties including gelling properties, emulsifying properties, whipping ability, and water‐binding capacity (Dissanayake, Ramchandran, Donkor, & Vasiljevic, 2013; Nicolai, Britten, & Schmitt, 2011; Singh, Oiseth, Lundin, & Day, 2014; Souza et al., 2012). The functionalities of WP can be enhanced or modified with certain strategies to meet different requirements in a wide range of food applications (Koo, Chung, Ogren, & Mutilangi, 2018; Onwulata, 2008).

Thermal treatment is one of the most commonly utilized processing methods that has greatly expanded the application of WP in food‐related products (Chung, Degner, & Mcclements, 2014; Famelart, Schong, & Croguennec, 2018; Torres, Janh, Mikkelsen, & Ipsen, 2011; Wang, Bao, Hendricks, & Guo, 2012; Zhang, McCarthy, Wang, Liu, & Guo, 2015). Thermal treatment above denaturation temperature (50–75°C) results in unfolding of the compact structure of WP, exposure of the buried apolar groups of polypeptide chains and sulfhydryl/disulfide exchange chain reactions via activated thiol groups (Dissanayake, Ramchandran, Piyadasa, & Vasiljevic, 2013; Guyomarc'h et al., 2014). WP, mainly α‐lactalbumin (α‐La) and β‐lactoglobulin (β‐Lg), forms aggregates cross‐linked by intermolecular disulfide bonds and noncovalent interactions. The aggregates are maintained by the unfolded α‐La and β‐Lg over the hydrophobic residues and the presence of thiol and disulfide groups (Nicolai et al., 2011).

The functional properties of WP depend on the internal arrangement, the properties of the surface charge and hydrophobicity, and the shape and size of protein particles. Thermal treatment changes aggregate size, the degree of which depends on the protein concentration, pH, ionic type, and heating temperature (Dissanayake, Ramchandran, Donkor, et al., 2013; Fachin & Viotto, 2005; Fitzsimons, Mulvihill, & Morris, 2007; Homer, Lundin, & Dunstan, 2018; Sağlam, Venema, Devries, Shi, & Vanderlinden, 2013). Controlling the protein particle size is one of the strategies in the development of novel protein products (Sağlam et al., 2013). For example, the WP aggregate particle size has great impact on the viscosity (Aziznia, Khosrowshahi, Madadlou, Rahimi, & Abbasi, 2009), the smoothness and creamy perception of yogurts (Krzeminski, Großhable, & Hinrichs, 2011; Protte, Weiss, & Hinrichs, 2018), and lightness of semisolid food emulsions (Chung et al., 2014). Therefore, manipulation of protein particles (including the size and size distribution) is important for food processing (Sağlam et al., 2013).

Sodium triphosphate (Na_5_P_3_O_10_) is recognized as safe and permitted to use as a food additive (the permitted level was <0.4%, w/w) by the U.S. Food and Drug Administration. The phosphate residues of Na_5_P_3_O_10_ can be attached to the ε‐amino groups of lysine or the side‐chain amino groups of imidazole or arginine (Woo, Creamer, & Richardson, 1982). Whey protein–sodium triphosphate aggregate addition can improve yogurt texture as a thickening agent (Cheng et al., 2017). Meanwhile, WP aggregates of different sizes could be manipulated by heating whey protein concentrate (WPC) solutions with Na_5_P_3_O_10_. Because WPC is an inexpensive by‐product during cheese making, it is promising to utilize WPC to obtain aggregates with optimal particle size for certain food applications, for example, yogurt making (Cheng et al., 2017; Zhang et al., 2015). Therefore, in this study, we attempted to investigate the effects of whey protein concentration, Na_5_P_3_O_10_ concentration, pH, heating temperature, and time on particle size and size distribution of WPC aggregates.

## 2 MATERIALS AND METHODS

### 2.1 Preparation of WPC aggregates

Whey protein concentrate powder (WPC 8000; Hilmar ingredients, California, USA) in this study contained 81.4% of protein (dry basis), 5.3% of moisture, 4.9% of fat, 5.2% of lactose, 3.2% of ash, 0.175% of sodium, 0.55% of calcium, 0.53% of potassium, 0.35% of phosphorus, 0.06% of magnesium, 0.125% of chloride, and 0.001% of iron. The WPC was reconstituted to achieve solutions with different protein concentrations (8.0%–10%, w/v), and then stored in a 4°C refrigerator for hydration for 12 hr. Na_5_P_3_O_10_ (Analytical reagent; Tianjin East China Chemical Reagent Factory, China) was added to the WPC solution and then stirred for at least 15 min. The solution was warmed up to room temperature, and its pH was adjusted to 7.5–8.5 with 2 mol/L NaOH, and then heated at 70–85°C for 5–25 min with continuous stirring.

### 2.2 Particle size determination

The particle size as well as its distribution of WPC aggregates was determined using a Mastersizer 2000 (Malvern Instruments Ltd, UK) based on the Mie theory within a range of 0.02–2,000 μm.

### 2.3 Transmission electron microscopy (TEM)

The microstructure of the WPC‐Na_5_P_3_O_10_ aggregates (protein content, 8%; Na_5_P_3_O_10_ content, 0.4%; pH 7.5; heated at 85°C for 5 min), WPC aggregates (protein content, 8%; Na_5_P_3_O_10_ content, 0%; pH 7.5; heated at 85°C for 5 min), and untreated whey protein solutions (protein content, 8%; pH 7.5) were examined by TEM (Walsh, Ross, Hendricks, & Guo, 2010). A drop of the WPC aggregates or whey protein solution was 1,000‐fold diluted in deionized water and then loaded onto a Formvar–carbon‐coated copper grid, individually. A droplet of 1% phosphotungstic acid at pH 7.0 was added and kept for 15 s before removing any excess. The grid was dried at room temperature and then observed using a transmission electron microscope (Hitachi‐600; Hitachi, Japan) operated at 80 kV. The images were captured automatically.

### 2.4 Statistical analysis

Statistical analyses were conducted using SPSS 11.5 or Origin 8.0, while the graphs were plotted using Malvern Mastersizer software. All the data were presented as the mean ± *SD* (*n* = 3).

## 3 RESULTS AND DISCUSSION

### 3.1 Effect of protein content on the WPC aggregates particle size

**Figure 1:**
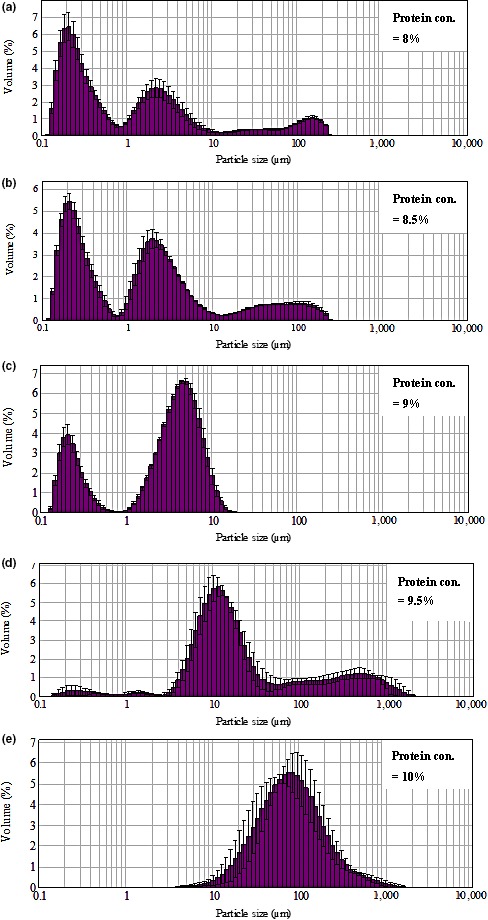
Effect of protein content on WPC‐Na_5_P_3_O_10_ aggregates particle size distribution (a–e: the content of Na_5_P_3_O_10_ 0.5%, pH 7.5, heated at 85°C for 5min, with the protein content of a, 8%; b, 8.5%; C, 9%; d, 9.5%; e, 10%). WPC, whey protein concentrate

When the protein content was 9%, the particle size was at bimodal distribution (Figure 1c). When over 54.18% WPC aggregates was within the range of 3.1–10 μm in size, few large particles (>10 μm) were detected. When the protein content was increased to 9.5%, the number of small particles (<1 μm) decreased nearly to zero, and the particles in the range of 1–10 μm increased to maximum (Figure 1a–d). When the protein content was equal or greater than 9.5%, the particle size was at a unimodal distribution, and more than 60% WPC aggregates were larger than 10 μm in size.

The heat‐induced protein aggregation is mainly attributable to formation of the disulfide‐bonding during aggregation (Krzeminski, Prell, Weiss, & Hinrichs, 2014). Almost all the β‐lactoglobulins (~60% of whey protein) were incorporated into the polymers via disulfide bonds, whereas α‐lactalbumins (~20% of whey protein) were bonded via hydrophobic interactions to a considerable extent (Fuente, Singh, & Hemar, 2002; Krzeminski et al., 2014; Mehalebi, Nicolai, & Durand, 2008; Nicolai et al., 2011; Zhang & Vardhanabhuti, 2014). Increased protein content provides more reactive sites that are able to link with the phosphate groups. Excessive whey proteins may polymerize by themselves during heat treatment. The protein content mainly affects protein aggregation instead of protein unfolding (Dissanayake, Ramchandran, Donkor, et al., 2013; Wolz, Mersch, & Kulozik, 2016). At a low concentration, the oligomers keep in a steady state and just few large particles can be formed via collision induced aggregation (Protte et al., 2018), while a higher concentration leads to formation of large aggregates (Sağlam et al., 2013).

### 3.2 Effect of Na_5_P_3_O_10_ content on the WPC aggregates particle size

**Figure 2:**
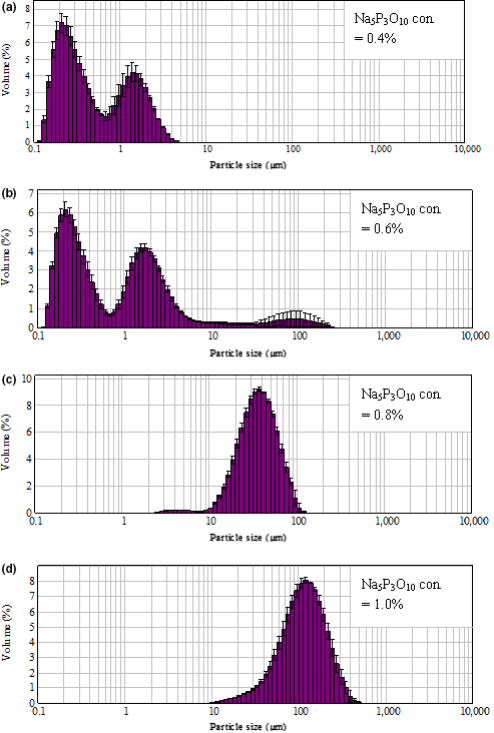
Effect of Na_5_P_3_O_10_ content on WPC‐Na_5_P_3_O_10_ aggregates particle size distribution (the content of protein 9%, pH 7.5, heated at 85°C for 5 min with the Na_5_P_3_O_10_ content of a, 0.4%; b, 0.6%; c, 0.8%; d, 1.0%). WPC, whey protein concentrate

With Na_5_P_3_O_10_ increasing from 0.4% to 0.6%, the small particles (<1 μm) and medium small‐sized particles (1–3 μm) reduced, while the medium‐sized particles (3.1–10 μm) and large particles (>10 μm) increased. However, when the Na_5_P_3_O_10_ content was over 0.8%, nearly all particles were larger than 10 μm with increased viscosity (Figure 2). The WPC aggregate solution was too viscous for particle size measurement when the Na_5_P_3_O_10_ was over 1.2%. When Na_5_P_3_O_10_ was at 0.4%, the particle size was in a bimodal distribution. When Na_5_P_3_O_10_ increased to 0.6%, the large particles (>10 μm) began to appear in a bimodal distribution. When Na_5_P_3_O_10_ content was greater than 0.8%, the particle size turned to display a unimodal distribution (Figure 2).

Phosphate group reacts with the active groups of protein side chains, such as the –OH of serine and threonine, and the ε‐NH_2_ of lysine (Woo et al., 1982). The phosphorus content of phosphorylated poly‐L‐lysine was higher than that of other common amino acids, indicating the reaction between phosphate group and protein was greatly affected by the content of L‐lysine in protein composition (Li et al., 2009). When the level of Na_5_P_3_O_10_ increased, the superfluous‐free Na_5_P_3_O_10_ probably participated in protein aggregation as salts. Salts (usually NaCl or CaCl_2_) can beaded to whey protein solutions to form aggregates with different morphologies and sizes, suggesting that the net charge of the native proteins (tunable by pH or ionic strength) is a crucial parameter for the formation of spherical particles. Increasing the ionic strength leads to charge screening and hence widens the pH range over which spherical particles can form (Baussay, Bon, Nicolai, Durand, & Busnel, 2004; Guyomarc'h et al., 2014; Homer et al., 2018; Mercad‐Prieto, Paterson, & Wilson, 2009; Phan‐Xuan et al., 2013; Pouzot, Durand, & Nicolai, 2004).

### 3.3 Effect of heating temperature and time on WPC aggregates particle size

**Figure 3:**
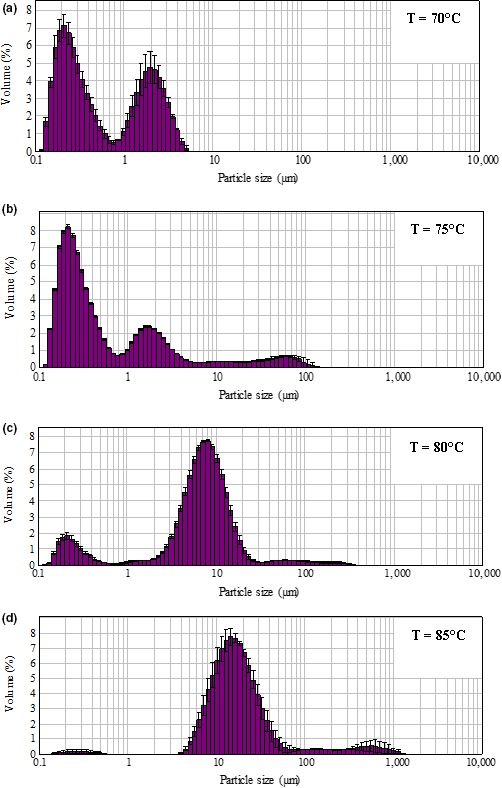
Effect of heating temperature on WPC‐Na_5_P_3_O_10_ aggregates particle size distribution (the content of protein 9%, and Na_5_P_3_O_10_ 0.5%, pH 7.5, heated for 15 min at a, 70°C; b, 75°C; c, 80°C; d, 85°C). WPC, whey protein concentrate

**Figure 4:**
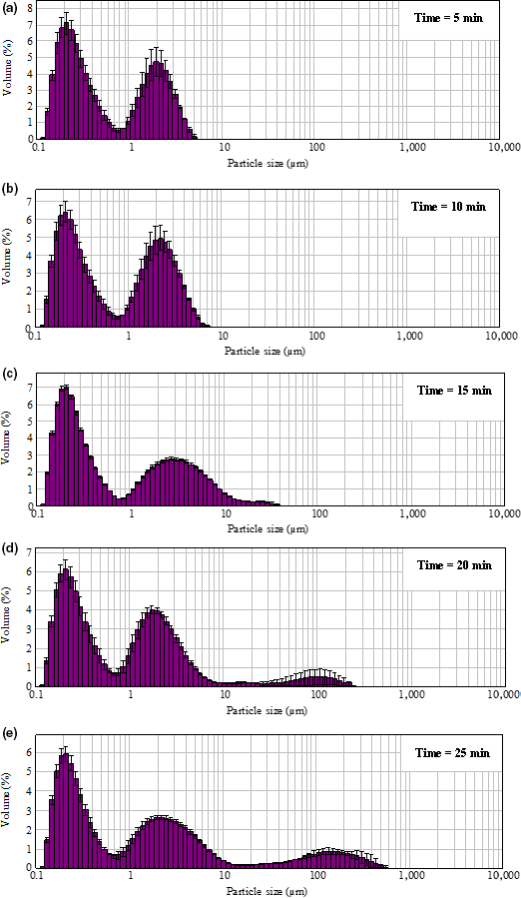
Effect of heating time on WPC‐Na_5_P_3_O_10_ aggregates particle size distribution (a–e: the content of protein 9% and Na_5_P_3_O_10_ 0.5%, pH 7.5, heated at 75°C for a, 5 min; b, 10 min; c, 15 min; d, 20 min; e, 25 min). WPC, whey protein concentrate

When the temperature increased from 70 to 85°C, the small (<1 μm) and medium small‐sized particles (1–3 μm) reduced, while the medium sized (3.1–10 μm) and the large particles (>10 μm) increased. The peak of particle size distribution was moved from small size region (approximately 1 μm) (Figure 3a and b) toward the medium size region (Figure 3c), and then to the large size region (Figure 3d) along with increasing temperature. When the heating time was prolonged, the percentage of small particles (<1 μm) decreased from 68.0% to 25.6%, and the medium small‐sized particles (1–3 μm) decreased from 26.5% to 15.0%. Heating for over 15 min generated more than 20% of large particles (>10 μm) (Figure 4).

Heating time and temperature are two major factors that affect the degree of protein aggregation. The higher temperature and longer heating time, the higher degree of aggregation. At the beginning, the smaller particles were limited by Brownian motion (Wolz et al., 2016), as a result, the average particle size was nearly unchanged. The growth of aggregates occurred with increasing heating time, resulting in the increase in particle size (Dissanayake, Ramchandran, Donkor, et al., 2013).

Increased temperature (from 35 to 85°C) accelerates the collision frequency between the amino acids and the phosphates (Ibrahim, Kobayashi, & Kato, 2014). Heating β‐lactoglobulin at 65°C can produce particles of a constant average size. However, when the temperature reaches up to 90°C, further increase in the heating temperature can result in only small increase in the rate of denaturation, as aggregation of the proteins becomes rate‐limiting (Gulzar, Bouhallab, Jeantet, Schuck, & Croguennec, 2011; Ibrahim et al., 2014).

### 3.4 Effect of pH on WPC aggregates particle size distribution

**Figure 5:**
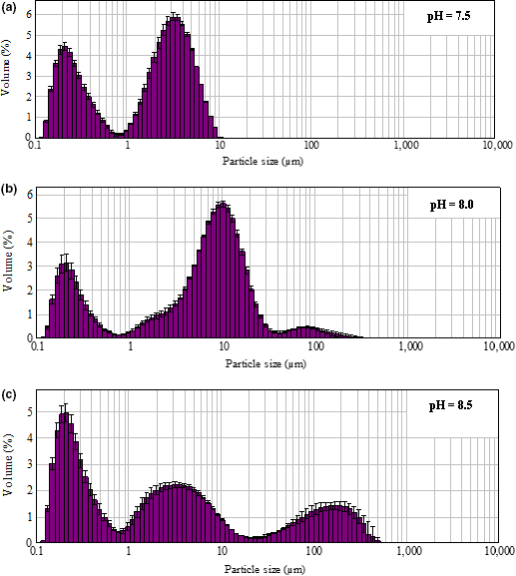
Effect of pH on WPC‐Na_5_P_3_O_10_ aggregates particle size distribution (the content of protein 9%, and Na_5_P_3_O_10_ 0.5%, heated at 85°C for 5 min under the pH a, 7.5; b, 8.0; c, 8.5). WPC, whey protein concentrate

Stable aggregates occur at pH values between 5.8 and 9.0 (Mehalebi et al., 2008). The β‐Lactoglobulin exhibits pH‐dependent reversible self‐association behavior (Yan et al., 2013) and usually exists in monomer–dimer equilibrium, which means the aggregation and dissociation may exist at the same condition. At pH between 5.5 and 7.5, a stable noncovalent dimer was formed (Mehalebi et al., 2008), when the pH was 7.5, the particles with medium small size (1–3 μm) reached maximum in quantity (28.9%), and the large particles (>10 μm) were barely detected (Figure 5a). The continuous process where aggregates grow and intermolecular interactions occur leads to a continuous increase in the size of the whey protein aggregates. When the pH was 8.0, over 50% of the particles were larger than 10 μm, and the percentage of particles (1–3 μm) was less than 5% (Figure 5b). This was likely due to that protein aggregation as well as the rates and reaction orders was affected by pH. When pH was between 7–11, increasing the pH accelerated the aggregation (Mercade‐Prieto & Gunasekaran, 2009). As reported, the dimer starts to dissociate into monomers above pH 7.5 (Estevez et al., 2016), when the pH was adjusted to 8.5, the large polymers tended to dissociate into smaller aggregates, and the medium‐sized particles (3.1–10 μm) reached the maximum level (41.3%) (Figure 5c). At pH 7–9, activated‐OH and ε‐NH_2_ of amino acids were easily bound by phosphate groups (Xiong, Zhang, & Ma, 2016; Zhang, Li, & Ren, 2007). It is supposed that, the degree of the reaction between phosphate groups and proteins increased with increasing pH, however, the increased negative charges of protein hinder WP from forming larger aggregates.

### 3.5 Microstructure of the WPC aggregates

**Figure 6:**
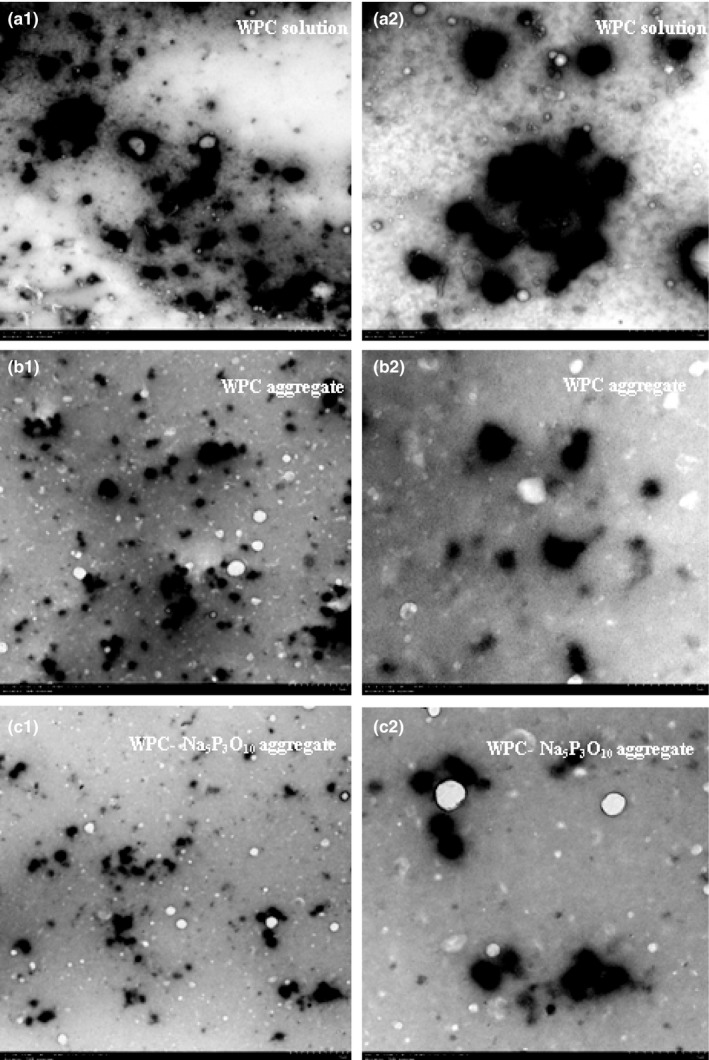
TEM micrographs of whey protein concentrate (WPC) solution (a), WPC aggregate (b), and WPC‐Na_5_P_3_O_10_ aggregates (c). Left (a_1_, b_1_, c_1_): magnification is 1,500×, scale bar: 2 μm; right (a_2_, b_2_, c_2_): magnification is 4,000×, scale bar: 1 μm (the bright spots are bubbles or voids; the dark imagines represent proteins aggregates). TEM, transmission electron microscopy

The whey protein particles in the WPC solution were close to each other (Figure 6a_1_–a_2_). When the whey protein was heated at 85°C for 5 min, the particles tended to disperse (Figure 6b_1_), and their shape turned irregular (Figure 6b_2_). The WPC‐Na_5_P_3_O_10_ aggregates showed a more even distribution (Figure 6c_1_) than that of either WPC solution (Figure 6a_1_) or the WPC aggregates (Figure 6b_1_). Additionally, the WPC‐Na_5_P_3_O_10_ aggregates approximated to homogeneous spherical particles (Figure 6c_2_).

It has been proposed that fractal aggregates resulted from self‐aggregation are dominant at higher protein concentrations (Fuente et al., 2002; Guyomarc'h et al., 2014; Nicolai et al., 2011). However, most of the particles in the WPC‐Na_5_P_3_O_10_ aggregates were not fractal aggregates as reported (Dissanayake, Ramchandran, Piyadasa, et al., 2013; Nicolai et al., 2011; Phan‐Xuan et al., 2013), but their particles were more regular and smoother as spheres than that in WPC aggregates (Figure 6b_2_). Aggregates formed by coacervation of electrostatic‐associated proteins are susceptible to changes in the ionic environment and/or net charge. Phosphate groups linked on amino acids changed the net charge of proteins so that WP hardly formed networks, but kept dispersed in solutions. The density of the aggregates decreased due to the increase in electrostatic repulsions between them. Na_5_P_3_O_10_ tuned the dimensions of the whey protein assemblies leading to the change of shape and size by increased surface hydrophobicity of WP. The buried hydrophobic residues became more exposed to the surface of protein by repulsion of negative electrostatic force of introduced phosphate groups (Li et al., 2009).

Manipulation of the particle size can potentiate whey protein in different applications. For example, the fat globules usually exist with average size of 3 μm in raw milk and reduce to approximately 1 μm in size after homogenization (Chung et al., 2014). From this point, the desirable particle size of a fat replacer should be within 1–3 μm. In fact, the fat replacer with the size less than 10 μm can give the sense of smoothness and consistency similar to the milk fat (Ertekin & Guzel‐Seydim, 2010). Therefore, the protein content should be controlled below 9% to assure most of the WPC aggregates within the range of 1–3 μm after heat treatment. From our experiment, the desirable sized particles can only be obtained when higher temperature matched shorter heating time and lower temperature matched longer heating time.

## 4 CONCLUSIONS

It is possible to prepare different sizes of WPC aggregates by manipulating the processing conditions such as protein content and Na_5_P_3_O_10_ level, pH, heating temperature, and time. Thermal treatment at controlled conditions can produce the majority of the aggregates as spherical particles of 1–3 μm in diameter that is suitable for use as a fat replacer in non‐ or low‐fat dairy product formulations.

## CONFLICT OF INTEREST

The authors declare that they have no conflict of interests.

## ETHICAL REVIEW

This study does not involve any human or animal testing.
